# Selected Polymorphisms of Base Excision Repair Genes and Pancreatic Cancer Risk in Japanese

**DOI:** 10.2188/jea.JE20120010

**Published:** 2012-11-05

**Authors:** Makoto Nakao, Satoyo Hosono, Hidemi Ito, Miki Watanabe, Nobumasa Mizuno, Shigeki Sato, Yasushi Yatabe, Kenji Yamao, Ryuzo Ueda, Kazuo Tajima, Hideo Tanaka, Keitaro Matsuo

**Affiliations:** 1 Division of Epidemiology and Prevention, Aichi Cancer Center Research Institute, Nagoya, Japan; 1 愛知県がんセンター研究所疫学・予防部; 2 Department of Medical Oncology and Immunology, Nagoya City University Graduate School of Medical Sciences, Nagoya, Japan; 2 名古屋市立大学大学院医学研究科腫瘍・免疫内科学; 3 Department of Gastroenterology, Aichi Cancer Center Central Hospital, Nagoya, Japan; 3 愛知県がんセンター中央病院消化器内科部; 4 Department of Pathology and Molecular Diagnostics, Aichi Cancer Center Central Hospital, Nagoya, Japan; 4 愛知県がんセンター遺伝子病理診断部; 5 Department of Epidemiology, Nagoya University Graduate School of Medicine, Nagoya, Japan; 5 名古屋大学大学院医学研究科健康社会医学専攻疫学

**Keywords:** pancreatic cancer, SNPs, DNA repair gene, *XRCC1*

## Abstract

**Background:**

Although several reports have described a possible association between DNA repair genes and pancreatic cancer (PC) in smokers, this association has not been fully evaluated in an Asian population. We assessed the impact of genetic polymorphisms in the base excision repair (BER) pathway on PC risk among Japanese.

**Methods:**

This case-control study compared the frequency of 5 single-nucleotide polymorphisms (SNPs) of BER genes, namely rs1052133 in *OGG1*, rs1799782 and rs25487 in *XRCC1*, rs1130409 in *APE1*, and rs1136410 in *PARP1*. SNPs were investigated using the TaqMan assay in 185 PC cases and 1465 controls. Associations of PC risk with genetic polymorphisms and gene–environment interaction were examined with an unconditional logistic regression model. Exposure to risk factors was assessed from the results of a self-administered questionnaire. We also performed haplotype-based analysis.

**Results:**

We observed that the minor allele of rs25487 in *XRCC1* was significantly associated with PC risk in the per-allele model (odds ratio = 1.29, CI = 1.01–1.65; trend *P* = 0.043). Haplotype analysis of *XRCC1* also showed a statistically significant association with PC risk. No statistically significant interaction between *XRCC1* polymorphisms and smoking status was seen.

**Conclusions:**

Our findings suggest that *XRCC1* polymorphisms affect PC risk in Japanese.

## Abstract (ja)

**【背景】:**

喫煙者においてDNA修復遺伝子と膵臓がんリスクの関連性がいくつかの研究で報告されているが、アジア人集団においてはまだ十分に検討されていない。本研究では、塩基除去修復系の遺伝子多型が膵臓がんリスクに与える影響について日本人集団で検討した。

**【方法】:**

塩基除去修復系遺伝子の5個の一塩基多型（SNPs）、rs1052133（OGG1）、rs1799782およびrs25487（XRCC1）、rs1130409（APE1）、rs11364101（PARP1）、について症例対照研究で検討した。185人の膵臓がん患者と1465人の非がん患者において、TaqMan法によるSNPs解析を行った。膵臓がんリスクと遺伝子多型および遺伝子‐環境交互作用については、unconditional logistic解析で評価した。またハプロタイプに基づいた解析も行った。

**【結果】:**

rs25487（XRCC1）のマイナーアレルは、per-alleleモデルにおいて、有意に膵臓がんリスクと関連していた（オッズ比：1.29、95％CI：1.01-1.65；trend P=0.043）。XRCC1のハプロタイプ解析においても、膵臓がんリスクと統計学的に有意な関連を認めた。XRCC1遺伝子多型と喫煙状況については、統計学的に有意な交互作用を認めなかった。

**【結論】:**

XRCC1遺伝子多型が、日本人集団において膵臓がんリスクに影響を与えることが示唆された。

## INTRODUCTION

The incidence of pancreatic cancer (PC) is increasing, and PC is now the fifth leading cause of cancer death in Japan.^1^^–^^5^ Because early detection and curative treatment of PC are very difficult, the 5-year survival rate is only 5.5%.^6^ This suggests that epidemiologic approaches to identifying PC high-risk groups have an important role in decreasing the number of PC deaths.

Possible risk factors for PC include advanced age, smoking, overweight, diabetes mellitus, and alcohol consumption.^2^^,^^7^^–^^10^ A positive association between PC risk and a family history of PC has also been reported, suggesting the possible involvement of genetic factors in PC incidence.^5^^,^^10^^,^^11^ Recently, several reports have observed an association between polymorphisms in DNA repair genes and PC risk, particularly among smokers.^12^^–^^20^ Four major types of DNA repair system have been identified, namely base excision repair (BER), nucleotide excision repair, mismatch repair, and double-strand break repair.^14^^,^^21^^–^^23^ With regard to the association between PC risk and DNA repair genes, genetic polymorphisms in the BER pathway are the most extensively investigated in molecular epidemiologic studies.^14^ However, most studies of the association between PC risk and BER gene polymorphisms have been conducted in white populations.

We conducted a case-control study in a Japanese population to evaluate the impact of genetic polymorphisms in BER pathways on PC risk among Asians. The study focused on 4 key genes in this repair pathway: X-ray repair cross-complementing group 1 (*XRCC1*), apurinic/apyrimidinic endonuclease (*APE1*), 8-oxoguanine DNA glycosylase (*OGG1*), and poly(ADP-ribose) polymerase 1 (*PARP1*).

## METHODS

### Study subjects

The case subjects were 185 PC patients with no prior history of cancer who were diagnosed at Aichi Cancer Center Hospital (ACCH), Nagoya, Japan, between January 2001 and November 2005. The control subjects comprised 1465 randomly selected non-cancer ACCH outpatients, during the same period, who had no history of any cancer. All subjects were enrolled in the Hospital-based Epidemiological Research Program II at ACCH (HERPACC-II) at the time of their first visit to ACCH. The framework of HERPACC-II has been described elsewhere.^24^^,^^25^ Briefly, all first-visit ACCH outpatients aged 20 to 79 years were asked to complete a self-administered questionnaire on their lifestyle before development of the presenting symptoms. Outpatients were also asked to provide a 7-mL blood sample. Approximately 95% of eligible subjects completed the questionnaire, and 50% provided blood samples. All data were loaded into the HERPACC database, which is periodically synchronized with the hospital cancer registry system. Approximately 35% of subjects were diagnosed with cancer within a year of the first visit. In the present study, we defined the case population as patients who received a diagnosis of PC within 1 year of the first visit, ie, we used the window period from first visit to final diagnosis of PC rather than prospectively identifying cases. A total of 75.7% of PC cases were histologically confirmed, among which 92.1% were diagnosed as having ductal adenocarcinoma. Our previous study showed that the lifestyle patterns of first-visit outpatients corresponded with those of individuals randomly selected from Nagoya’s general population, which confirms the external validity of the study.^26^ The present study was approved by the Ethics Committee of Aichi Cancer Center, and informed consent was obtained at first visit from all participants.

### Genotyping

We examined 5 non-synonymous polymorphisms in BER pathway genes, namely rs1799782 (Arg194Trp) and rs25487 (Arg399Gln) in *XRCC1*, rs1130409 (Asp148Glu) in *APE1*, rs1052133 in *OGG1* (Ser326Cys), and rs1136410 (Val762Ala) in *PARP1*. In our study, these 5 single-nucleotide polymorphisms (SNPs) were selected on the basis of their association with cancer risk or their expected influence on BER systems.^13^^,^^14^^,^^17^^–^^22^^,^^27^^–^^29^ DNA of each subject was extracted from the buffy coat fraction using a DNA Blood Mini Kit (Qiagen, Tokyo, Japan). All loci were examined by the TaqMan assay with probes and primers (Applied Biosystems, Foster City, CA, USA) and the Fluidigm EP1 SNP Genotyping 96.96 Dynamic Array (Fluidigm Corp., South San Francisco, CA, USA). Approximately 10% of subjects were examined in duplicate to confirm the consistency of genotyping.

### Assessment of exposure

Exposure to potential PC risk factors was assessed from responses to the self-administered questionnaire, which was completed before diagnosis during the first visit to ACCH and reviewed by trained interviewers. Subjects were specifically questioned about their lifestyle before the onset of presenting symptoms. Daily alcohol consumption in grams was determined by summing the amount of pure alcohol in the average daily consumption of Japanese *sake* (rice wine), *shochu* (distilled spirit), beer, wine, and whiskey. Cumulative smoking exposure was measured in pack-years (PYs), ie, the product of the average number of packs per day and the number of years of smoking. Height and body weight at baseline and weight at age 20 years were self-reported. Current body mass index (BMI) and BMI at age 20 were calculated by dividing the weight in kilograms by the height in meters squared. Family history of PC was considered positive when at least 1 parent or sibling had a history of PC.

### Statistical analysis

All statistical analyses were performed using Stata version 10 (Stata Corp., College Station, TX, USA). A *P*-value less than 0.05 was considered statistically significant. Differences in characteristics between cases and controls were assessed using the chi-square test. Odds ratios (ORs) and 95% CIs were estimated using an unconditional logistic regression model adjusted for potential confounders. Potential confounders considered in multivariate analysis were age, sex, PYs of smoking (<5, ≥5 but <20, ≥20 but <40, or ≥40), drinking habit (nondrinker, <23, ≥23 but <46, or ≥46 g/day), current BMI (<18.5, ≥18.5 but <22.5, ≥22.5 but <25, ≥25 but <27.5, or ≥27.5 kg/m^2^), BMI at age 20 (<18.5, ≥18.5 but <22.5, ≥22.5 but <25, ≥25 but <27.5, or ≥27.5 kg/m^2^), history of diabetes mellitus (yes or no), and family history of PC (yes or no). Interactions between environmental factors and genotypes were assessed by using likelihood ratio tests within logistic regression models, with and without interaction terms. In haplotype analysis, we used haplotype-effects logistic regression for case-control data.^30^ This haplotype analysis uses phased and unphased SNP genotype data to estimate haplotype effects and haplotype–environment interactions in a case-control study.^30^^,^^31^ It fits haplotype-effects logistic regression by using the retrospective likelihood method in a special case of a rare disease and a single candidate gene in the Hardy–Weinberg Equilibrium (HWE), under the assumption of gene–environment independence.^30^^,^^31^ Accordance with the HWE was assessed by the chi-square test.

## RESULTS

The background characteristics of the subjects are shown in Table 1. Men accounted for 68.7% of case subjects and 74.9% of controls. As compared with the control group, the case group had a significantly higher prevalence of heavy smokers (*P* = 0.005), a higher prevalence of a history of diabetes mellitus (*P* < 0.001), a lower current BMI (*P* = 0.021), and a higher BMI at age 20 (*P* = 0.018).

**Table 1.** Comparison of selected characteristics of pancreatic cancer (PC) patients and non-cancer controls

	Cases (%)	Controls (%)	*P*-values^a^
		
	*n* = 185	*n* = 1465	
Age			0.999
<40	10 (5.41)	75 (5.12)	
≥40 but <50	19 (10.27)	147 (10.03)	
≥50 but <60	60 (32.43)	479 (32.70)	
≥60 but <70	60 (32.43)	484 (33.04)	
≥70	36 (19.46)	280 (19.11)	
Sex			0.068
Male	127 (68.65)	1097 (74.88)	
Female	58 (31.35)	368 (25.12)	
Current BMI^b^ (kg/m^2^)			0.021
<18.5	15 (8.11)	61 (4.16)	
≥18.5 but <22.5	84 (45.11)	555 (37.88)	
≥22.5 but <25	47 (25.41)	478 (32.63)	
≥25 but <27.5	25 (13.51)	245 (16.72)	
≥27.5	14 (7.57)	111 (7.58)	
Unknown	0 (0.00)	15 (1.02)	
BMI^b^ at age 20 (kg/m^2^)			0.018
<18.5	14 (7.57)	168 (11.47)	
≥18.5 but <22.5	112 (60.54)	950 (64.85)	
≥22.5 but <25	36 (19.46)	226 (15.43)	
≥25 but <27.5	11 (5.95)	72 (4.91)	
≥27.5	6 (3.24)	14 (0.96)	
Unknown	6 (3.24)	35 (2.39)	
Cigarette pack-years			0.005
<5	69 (37.30)	638 (43.55)	
≥5 but <20	22 (11.89)	206 (14.06)	
≥20 but <40	34 (18.38)	308 (21.02)	
≥40	60 (32.43)	304 (20.75)	
Unknown	0 (0.00)	9 (0.61)	
Drinking, g ethanol/day			0.568
Non	56 (30.27)	488 (33.31)	
<23	53 (28.65)	425 (29.01)	
≥23 but <46	43 (23.24)	342 (23.34)	
≥46	31 (16.76)	193 (13.17)	
Unknown	2 (1.08)	17 (1.16)	
History of diabetes mellitus			<0.001
Yes	37 (20.00)	126 (8.60)	
No	148 (80.00)	1339 (91.40)	
Family history of PC			0.811
Yes	8 (4.32)	58 (3.96)	
No	177 (95.68)	1407 (96.04)	

^a^ *P*-values calculated by chi-square test.

^b^BMI: body mass index.

Table 2 shows genotype distributions for the 5 SNPs (rs1052133, rs1799782, rs25487, rs1130409, and rs1136410). Three of the 5 SNPs (rs1799782, rs25487, and rs1130409) were in accordance with HWE. The remaining 2 loci (rs1052133 and rs1136410) were not and were thus excluded from further analysis. Rs25487 in *XRCC1* was significantly associated with PC risk in the per-allele model (OR = 1.29, CI = 1.01–1.65; trend *P* = 0.043) and in the dominant model (OR = 1.39, CI = 1.01–1.91; *P* = 0.041). No significant association with PC risk was observed for the remaining 2 loci.

**Table 2.** Distribution of cases and controls, and odds ratios for pancreatic cancer associated with selected BER gene polymorphisms

Polymorphism	Genotype	No. of cases/controls (%)	Unadjusted ORs^a^ (95% CI)	Adjusted ORs^b^ (95% CI)
rs1799782	CC	88(47.57)/677(46.21)	1.00 (ref.)	1.00 (ref.)
(XRCC1, Arg194Trp)	CT	80(43.24)/636(43.41)	0.97 (0.70–1.33)	0.96 (0.69–1.34)
	TT	17(9.19)/152(10.38)	0.86 (0.50–1.49)	0.81 (0.46–1.44)
	Per-allele model		0.94 (0.75–1.19)	0.96 (0.75–1.21)
	Dominant model		0.95 (0.70–1.29)	0.93 (0.68–1.28)
	(*P* trend)		0.622	0.709
	minor allele(T) frequency in control subjects = 0.321 (HWE^c^: *P* = NS)			

rs25487	GG	93(50.27)/842(57.47)	1.00 (ref.)	1.00 (ref.)
(XRCC1, Arg399Gln)	GA	77(41.62)/538(36.72)	1.30 (0.94–1.79)	1.36 (0.98–1.90)
	AA	15(8.11)/85(5.80)	1.60 (0.89–2.88)	1.58 (0.85–2.93)
	Per-allele model		1.28 (1.00–1.63)	1.29 (1.01–1.65)
	Dominant model		1.34 (0.98–1.82)	1.39 (1.01–1.92)
	(*P* trend)		0.046	0.043
	minor allele(A) frequency in control subjects = 0.242 (*P* = NS)			

rs1130409	TT	77(41.62)/542(37.00)	1.00 (ref.)	1.00 (ref.)
(APE1, Asp148Glu)	TG	75(41.62)/681(46.48)	0.78 (0.55–1.09)	0.77 (0.55–1.10)
	GG	33(17.84)/242(16.52)	0.96 (0.62–1.48)	1.02 (0.65–1.60)
	Per-allele model		0.94 (0.75–1.16)	0.96 (0.77–1.20)
	Dominant model		0.82 (0.60–1.12)	0.84 (0.61–1.15)
	(*P* trend)		0.548	0.745
	minor allele(T) frequency in control subjects = 0.398 (*P* = NS)			

rs1052133	CC	55(29.73)/417(28.46)	—	—
(OGG1, Ser326Cys)	CG	87(47.03)/692(47.24)	—	—
	GG	43(23.24)/356(24.30)	—	—
	Per-allele model		—	—
	Dominant model		—	—
	(*P* trend)		—	—
	minor allele(G) frequency in control subjects = 0.479 (*P* = 0.040)			

rs1136410	TT	61(32.97)/550(37.54)	—	—
(PARP1, Val762Ala)	TC	90(48.65)/657(44.85)	—	—
	CC	34(18.38)/258(17.61)	—	—
	Per-allele model		—	—
	Dominant model		—	—
	(*P* trend)		—	—
	minor allele(T) frequency in control subjects = 0.400 (*P* = 0.012)			

Abbreviation: OR, odds ratio.

^a^Unconditional logistic regression model (unadjusted).

^b^Unconditional logistic regression model adjusted for age, sex, current BMI, BMI at age 20, smoking status, drinking habit, history of diabetes mellitus, and family history of pancreatic cancer.

^c^HWE: Hardy–Weinberg Equilibrium.

We analyzed potential interactions of rs1799782 and rs25487 in *XRCC1* and rs1130409 in *APE1* with known risk factors for PC, such as smoking, alcohol consumption, overweight, diabetes mellitus, and family history of PC. Because of the low frequency of minor homozygous subjects, these subjects were combined with heterozygous subjects in this analysis. The exposure variables used were current and former BMI (BMI <25 or ≥25 kg/m^2^), alcohol consumption (<23 or ≥23 g ethanol/day), smoking (<5 or ≥5 PYs), heavy smoking (<40 or ≥40 PYs), history of diabetes mellitus (yes or no), and family history of PC (yes or no). There were no statistically significant interactions (eTable 1). We also analyzed interactions of rs1799782 and rs25487 in *XRCC1* and rs1130409 in *APE1* with smoking duration and intensity and detected no statistically significant interactions (eTable 2).

Table 3 shows the results of haplotype analysis for *XRCC1*. The *R*-squared (*R* ^2^) value between rs1799782 and rs25487 was 0.15. Haplotype CA in *XRCC1* was associated with a statistically significant increase in PC risk (OR = 1.32, CI = 1.01–1.71; *P* = 0.042) as compared with the most common haplotype, CG, in *XRCC1*. As shown in Table 4, we defined haplotype CA in *XRCC1* as a risk haplotype and analyzed the potential interactions between risk haplotype and known PC risk factors (smoking, alcohol consumption, overweight, diabetes mellitus, and family history of PC). The results showed significant interactions between a family history of PC and the risk haplotype in *XRCC1* (interaction *P* = 0.020).

**Table 3.** Haplotype frequencies of *XRCC1*, and odds ratios for pancreatic cancer associated with *XRCC1* haplotype

Haplotype	SNPs^a^		Haplotype frequency			Adjusted OR^b^ (cases/controls 185/1465)	*P*-value
	
	1 (C>T)	2 (G>A)	Overall	Cases	Controls		
1	C	G	0.439	0.433	0.442	1.00 (ref.)	
2	C	A	0.240	0.274	0.239	1.32 (1.01–1.71)	0.042
3	T	G	0.319	0.292	0.318	1.05 (0.81–1.36)	0.691
4	T	A	0.002	<0.001	0.002	N.E.	N.E.

Abbreviation: OR, odds ratio.

^a^SNP 1 is rs1799782; SNP 2 is rs25487.

^b^Haplotype-effects logistic regression for case-control data was used. Multivariable adjustment by age, sex, current BMI, BMI at age 20, smoking status, drinking habit, diabetes mellitus, and family history of PC.

**Table 4.** Gene-environment interaction between *XRCC1* haplotypes and selected risk factors for pancreatic cancer

Haplotype	Exposure	Current BMI^a^	BMI^a^ at age 20	Smoking^b^	Heavy Smoking^b^	Alcohol^c^	Diabetes mellitus^d^	Family history of PC^e^
non-CA^f^	(−)	1.00 (ref.)	1.00 (ref.)	1.00 (ref.)	1.00 (ref.)	1.00 (ref.)	1.00 (ref.)	1.00 (ref.)
CA^g^	(−)	1.37 (1.05–1.79)	1.35 (1.05–1.73)	1.64 (1.13–2.38)	1.43 (1.08–1.90)	1.30 (0.95–1.77)	1.26 (0.97–1.65)	1.20 (0.93–1.54)

non-CA^f^	(+)	1.00 (ref.)	1.00 (ref.)	1.00 (ref.)	1.00 (ref.)	1.00 (ref.)	1.00 (ref.)	1.00 (ref.)
CA^g^	(+)	1.01 (0.59–1.73)	1.12 (0.53–2.36)	1.09 (0.80–1.50)	1.02 (0.64–1.63)	1.24 (0.85–1.82)	1.42 (0.79–2.52)	4.68 (1.59–13.81)

interaction *P*		0.468	0.124	0.095	0.097	0.729	0.864	0.020

NOTE: All values expressed as odds ratio (95% CI). Haplotype-effects logistic regression for case-control data was used. Multivariable adjustment by age, sex, current BMI, BMI at age 20, smoking status, drinking habit, diabetes mellitus, and family history of PC. Interactions between environmental factors and genotypes were assessed by likelihood ratio tests between the logistic regression models, with and without interaction terms, between genes and environmental factors of interest.

^a^BMI <25 vs. ≥25.

^b^Smoking: pack-years <5 vs. ≥5; Heavy Smoking: pack-years <40 vs. ≥40.

^c^Alcohol: g ethanol/day <23 vs. ≥23.

^d^Diabetes mellitus: no vs. yes.

^e^Family history of pancreatic cancer: no vs. yes.

^f^Haplotype non-CA: other haplotypes (frequency > 0.01) except haplotype CA. Frequency in control subjects was 0.760.

^g^Haplotype CA: *XRCC1* SNPs at rs1799782 and rs25487. Frequency in control subjects was 0.239.

## DISCUSSION

In this case-control study, we found that the genetic polymorphism rs25487 in *XRCC1* was associated with increased risk of PC in a Japanese population. However, we did not detect any statistically significant interactions with smoking.

The BER pathway has a primary role in the repair of oxidative base lesions such as 8-hydroxyguanine, formamidopyrimidines, and 5-hydroxyuracil.^21^ Oxidative damage to DNA may lead to mutations that activate oncogenes or inactivate tumor suppressor genes and may eventually increase the probability of genetic alterations developing into neoplastic events.^21^ Sequence variants in BER genes are thought to modulate DNA repair capacity and are consequently suspected of being associated with altered cancer risk.^20^ With regard to the association between PC risk and DNA repair pathway, genetic polymorphisms in the BER pathway are the most widely studied in Western epidemiologic studies.^14^ To our knowledge, however, this is the first study to detect an association between *XRCC1* polymorphisms and the development of PC in an Asian population.

Two common variants of *XRCC1* are rs1799782 (in which T substitutes for C) and rs25487 (in which A substitutes for G), which lead to amino acid substitutions Arg194Trp and Arg399Gln, respectively.^32^ In particular, the minor allele (A) for rs25487, namely the 399Gln allele, was found to be associated with a higher frequency of glycophorin mutant, elevated DNA adduct levels, higher baseline sister chromatid exchange frequency, and increased sensitivity to ionizing radiation, all of which might be due to reduced BER function.^18^ In the present study, the minor allele of rs25487 in *XRCC1* was associated with elevated PC risk. Thus, our finding is consistent with the potential mechanisms described above.

It has been suggested that *XRCC1* gene polymorphisms are modulating factors for PC risk, particularly in smokers.^17^^–^^21^ A synergistic effect between these gene polymorphisms and tobacco smoking in relation to PC risk has been suspected.^14^ Oxidative DNA damage produced by smoking is expected to be repaired by the BER system, which includes the *XRCC1* gene.^33^ In this study, we observed a significant main effect of rs25487 in *XRCC1* but found no statistically significant interaction between *XRCC1* loci and smoking. Although the reason for this difference is unclear, the results may have been affected by the heterogeneity of study populations, differences in ethnicity, the small sample size, and potential confounders. On the other hand, the minor allele of rs25487 in *XRCC1* was associated with increased risk of PC among light but not heavy smokers. These findings are consistent with past reports, and possible explanations for the limited effect among heavy smokers include enhanced apoptosis at the time of cell division from heavy smoking and induction of DNA repair capacity in response to DNA damage.^20^^,^^21^

In addition, we found that haplotype CG in *XRCC1* had a higher impact in those with a family history of PC than in those without such a history. To our knowledge, this phenomenon has not been reported previously and might be explained by family history acting as a surrogate for interaction with known/unknown genetic susceptibility factors. We did not observe any interaction with the *ABO* genotype, which has been reported to have a significant association with PC risk (data not shown).^34^ Given our small study population, the finding of an interaction with family history may have been due to chance. Further study is required to duplicate our findings in a larger cohort.

Our study has several methodological issues that warrant discussion. First, the control population was selected from non-cancer patients at ACCH. It is reasonable to assume that this was the same population from which the case subjects were derived, which would bolster the internal validity of our study. Second, with regard to external validity, we previously showed that individuals selected randomly from our control population were similar to the general population of Nagoya City in terms of the exposure of interest.^26^ However, only 50% of patients agreed to the collection of a blood sample, and the characteristics of these participants might differ from those of the general population. Third, case-control studies have an intrinsic information bias. However, the HERPACC system is less prone to this bias than are typical hospital-based studies, as the data for all patients were collected before diagnosis. In addition, the results from our self-administered questionnaire may be inaccurate. However, any such misclassification would be nondifferential and would likely underestimate the causal association.^35^ Fourth, with regard to SNP analysis, because 2 SNPs (rs1052133 in *OGG1* and rs1136410 in *PARP1*) were not in accordance with HWE in our study, further studies that include these factors are required. Fifth, residual confounding by known and unknown risk factors might be present; given the small number of cases, our findings require replication in a larger study. Finally, our study was limited to a Japanese population, and the results cannot necessarily be extrapolated to other populations.

In summary, this case-control study showed a significant association between genetic polymorphisms in *XRCC1* and PC risk in a Japanese population. Further investigation of our findings in larger populations are warranted, and the biological mechanisms responsible for the association should be fully elucidated.

## ONLINE ONLY MATERIALS

eTables and the Japanese-language abstract for articles can be accessed by clicking on the tab labeled Supplementary materials at the journal website http://dx.doi.org/10.2188/jea.JE20120010.

eTables.

Abstract in Japanese.
