# Supplementary material for: Selected Polymorphisms of Base Excision Repair Genes and Pancreatic Cancer Risk in Japanese
Source: J Epidemiol. 2012 Nov 5;22(6):477–83. doi: 10.2188/jea.JE20120010 (PMC3798558; doi:10.2188/jea.JE20120010)
Supplement: eTables. [file je-22-477-s001.pdf]

eTable 1. Gene–environment interaction of *XRCC1* and *APE1* genotypes with selected risk factors for pancreatic cancer (PC)

Genotype	Exposure of each environmental factor	Current BMI*		BMI* at age 20		Smoking§		Heavy Smoking§		Alcohol‡		Diabetes Mellitus‖		Family history of PC††	
		No. of case/control	ORs (95% CI)	No. of case/control	ORs (95% CI)	No. of case/control	ORs (95% CI)	No. of case/control	ORs (95% CI)	No. of case/control	ORs (95% CI)	No. of case/control	ORs (95% CI)	No. of case/control	ORs (95% CI)
	(–)	146/1096	1.00 (ref.)	162/1344	1.00 (ref.)	69/638	1.00 (ref.)	91/844	1.00 (ref.)	106/913	1.00 (ref.)	148/1339	1.00 (ref.)	177/1407	1.00 (ref.)
	(+)	39/356	0.72 (0.48 - 1.07)	17/86	1.68 (0.93 - 3.04)	116/818	1.66 (1.10 - 2.52)	94/612	1.71 (1.16 - 2.51)	74/535	1.07 (0.49 - 2.32)	37/126	2.61 (1.69 - 4.04)	8/58	1.06 (0.49 - 2.31)
rs1799782															
CC	(–)	69/517	1.00 (ref.)	78/636	1.00 (ref.)	30/294	1.00 (ref.)	40/396	1.00 (ref.)	46/430	1.00 (ref.)	66/619	1.00 (ref.)	82/648	1.00 (ref.)
CT&TT	(–)	77/577	0.98 (0.68 - 1.39)	84/708	0.98 (0.70 - 1.37)	39/344	1.06 (0.63 - 1.77)	51/448	1.01 (0.69 - 1.48)	63/483	1.20 (0.79 - 1.82)	82/720	1.02 (0.72 - 1.45)	95/759	0.97 (0.70 - 1.34)
CC	(+)	19/153	0.79 (0.45 - 1.39)	7 / 28	2.23 (0.89 - 5.57)	58/375	1.84 (1.07 - 3.16)	48/273	2.43 (1.44 - 4.08)	41/239	1.74 (1.07 - 2.81)	22/58	3.40 (1.88 - 6.11)	6 / 29	1.52 (0.59 - 3.90)
CT&TT	(+)	20/203	0.64 (0.37 - 1.13)	10/58	1.40 (0.66 - 2.99)	58/443	1.60 (0.92 - 2.77)	46/339	1.83 (1.09 - 3.10)	33/296	1.14 (0.69 - 1.90)	15/68	1.97 (1.02 - 3.81)	2 / 29	0.54 (0.12 - 2.37)
interaction P			0.649		0.456		0.546		0.408		0.070		0.195		0.240
rs25487															
GG	(–)	73/636	1.00 (ref.)	78/772	1.00 (ref.)	31/363	1.00 (ref.)	44/486	1.00 (ref.)	55/522	1.00 (ref.)	73/765	1.00 (ref.)	91/807	1.00 (ref.)
GA&AA	(–)	73/458	1.47 (1.03 - 2.10)	84/572	1.43 (1.02 - 2.00)	38/275	1.66 (0.99 - 2.77)	47/358	1.61 (1.10 - 2.36)	54/391	1.35 (0.90 - 2.05)	75/574	1.35 (0.96 - 1.92)	86/600	1.32 (0.95 - 1.83)
GG	(+)	20/198	0.80 (0.47 - 1.39)	13/49	2.54 (1.26 - 5.11)	62/474	1.93 (1.13 - 3.29)	49/351	2.62 (1.60 - 4.28)	37/311	1.21 (0.76 - 1.95)	20/77	2.45 (1.36 - 4.42)	2/35	0.47 (0.11 - 2.06)
GA&AA	(+)	19/158	0.90 (0.51 - 1.58)	4/37	1.17 (0.39 - 3.54)	54/344	2.35 (1.37 - 4.02)	45/261	2.63 (1.51 - 4.57)	37/224	1.82 (1.12 - 2.93)	17/49	3.95 (2.09 - 7.47)	6 / 23	2.41 (0.92 - 6.29)
interaction P			0.490		0.069		0.354		0.183		0.765		0.691		0.112
rs1130409															
TT	(–)	67/417	1.00 (ref.)	69/504	1.00 (ref.)	25/230	1.00 (ref.)	34/307	1.00 (ref.)	48/339	1.00 (ref.)	61/498	1.00 (ref.)	74/517	1.00 (ref.)
TG&GG	(–)	79/677	0.74 (0.52 - 1.06)	93/840	0.82 (0.58 - 1.15)	44/408	1.06 (0.62 - 1.81)	57/537	0.87 (0.59 - 1.28)	61/574	0.77 (0.51 - 1.17)	87/841	0.84 (0.59 - 1.20)	103/890	0.81 (0.58 – 1.13)
TT	(+)	10/122	0.47 (0.23 - 0.96)	6 /27	1.62 (0.62 - 4.26)	52/308	2.08 (1.16 - 3.73)	43/231	2.28 (1.30 - 3.99)	28/198	1.14 (0.67 - 1.93)	16/44	2.68 (1.36 - 5.26)	3 /25	0.72 (0.21 - 2.53)
TG&GG	(+)	29/234	0.67 (0.41 - 1.11)	11/59	1.42 (0.67 - 2.99)	64/510	1.51 (0.86 - 2.68)	51/381	1.71 (1.02 - 2.85)	46/337	1.07 (0.67 - 1.71)	21/82	2.16 (1.21 - 3.86)	5/33	1.13 (0.41 - 3.07)
interaction P			0.112		0.911		0.264		0.672		0.559		0.925		0.420

NOTE—Odds ratio (OR) (95% CI): Unconditional logistic regression model adjusted for age, sex, current BMI, BMI at age 20, smoking status, drinking habit, diabetes mellitus, and family history of PC. Interactions between environmental factors and genotypes were assessed by likelihood ratio tests between the logistic regression models, with and without interaction terms, between genes and environmental factors of interest.

* BMI <25 vs. ≥ 25

§ Smoking: pack-years < 5 vs. ≥ 5; Heavy Smoking: pack-years < 40 vs. ≥ 40

‡ Alcohol: g ethanol/day < 23 vs. ≥23

‖ Diabetes Mellitus: no vs. yes

†† Family history of PC: no vs. yes

eTable 2. Smoking duration/intensity and the risk of pancreatic cancer (PC)

	No. of case/controls	Unadjusted OR†	Adjusted OR††	Gene-environmental interactions‡		
				rs1799782 (*XRCC1*)	rs25487 (*XRCC1*)	rs1130409 (*APE1*)
Smoking duration (years)						
never smoker	65/581	1.00 (ref.)	1.00 (ref.)			
>0 but <20	24/207	0.84 (0.52–1.36)	1.00 (0.58–1.73)			
≥20 but <30	23/187	1.12 (0.75–1.68)	1.58 (0.95–2.64)	P=0.446	P=0.867	P=0.518
≥30 but <40	36/279	1.63 (0.94–2.83)	2.11 (1.10–4.04)			
≥40	37/202	1.82 (1.15–2.88)	2.48 (1.40–4.37)			
Unknown	0/9					
Smoking intensity (cigarettes per day)						
never smoker	65/580	1.00 (ref.)	1.00 (ref.)			
>0 but <20	25/266	1.04 (0.63–1.70)	1.39 (0.78–2.48)			
≥20 but <30	44/350	1.10 (0.66–1.82)	1.34 (0.75–2.48)	P=0.351	P=0.713	P=0.444
≥30 but <40	19/104	1.15 (0.75–1.78)	1.42 (0.83–2.43)			
≥40	32/157	1.64 (1.06–2.53)	2.03 (1.17–3.52)			
Unknown	0/8					

†Unconditional logistic regression model (unadjusted).

††Unconditional logistic regression model adjusted for age, sex, current BMI, BMI at age 20, smoking status, drinking habit, diabetes mellitus, and family history of PC.

‡Interactions between environmental factors and genotypes were assessed by likelihood ratio tests between the logistic regression models, with and without interaction terms, between genes and environmental factors of interest. Genotype: major homozygous vs. heterozygous and minor homozygous. Environmental factors: Smoking duration: <20 years vs. ≥20 years, Smoking intensity: <20 cigarettes vs. ≥20 cigarettes. Analysis was adjusted for age, sex, current BMI, BMI at age 20, smoking status, drinking habit, diabetes mellitus, and family history of PC.
