# Supplementary material for: Selected Polymorphisms of Base Excision Repair Genes and Pancreatic Cancer Risk in Japanese
Source: J Epidemiol. 2012 Nov 5;22(6):477–83. doi: 10.2188/jea.JE20120010 (PMC3798558; doi:10.2188/jea.JE20120010)
Supplement: Abstract in Japanese. [file je-22-477-s002.pdf]

日本人集団における塩基除去修復遺伝子多型と膵臓がんリスクの検討

中尾心人[1,2]、細野覚代[1]、伊藤秀美[1]、渡邉美貴[1]、水野伸匡[3]佐藤滋樹[2]、谷田部恭[4]、山雄健次[3]、上田龍三[4]、田島和雄[1]、田中英夫[1,5]、松尾恵太郎[1,5]

[1] 愛知県がんセンター研究所疫学・予防部、[2] 名古屋市立大学大学院医学研究科腫瘍・免疫内科学、[3] 愛知県がんセンター中央病院消化器内科部、[4] 愛知県がんセンター遺伝子病理診断部、[5]名古屋大学大学院医学研究科健康社会医学専攻疫学

【背景】喫煙者において DNA 修復遺伝子と膵臓がんリスクの関連性がいくつかの研究で報告されているが、アジア人集団においてはまだ十分に検討されていない。本研究では、塩基除去修復系の遺伝子多型が膵臓がんリスクに与える影響について日本人集団で検討した。
【方法】塩基除去修復系遺伝子の 5 個の一塩基多型（SNPs）、rs1052133（OGG1）、rs1799782 および rs25487（XRCC1）、rs1130409（APE1）、rs11364101（PARP1）、について症例対照研究で検討した。185 人の膵臓がん患者と 1465 人の非がん患者において、TaqMan 法による SNPs 解析を行った。膵臓がんリスクと遺伝子多型および遺伝子 - 環境交互作用については、unconditional logistic 解析で評価した。またハプロタイプに基づいた解析も行った。
【結果】rs25487（XRCC1）のマイナーアレルは、per-allele モデルにおいて、有意に膵臓がんリスクと関連していた（オッズ比 : 1.29、95%CI : 1.01-1.65 ; trend P=0.043）。XRCC1 のハプロタイプ解析においても、膵臓がんリスクと統計学的に有意な関連を認めた。XRCC1 遺伝子多型と喫煙状況については、統計学的に有意な交互作用を認めなかった。
【結論】XRCC1 遺伝子多型が、日本人集団において膵臓がんリスクに影響を与えることが示唆された。

キーワード : 膵臓がん、SNPs、DNA 修復遺伝子、XRCC1
